# Designing a Drone Control Station for Team Missions with Educational Drones

**DOI:** 10.3390/s26041281

**Published:** 2026-02-16

**Authors:** Jessika Delgado, Bushra Younas, Jaeho Kim, Sungsoo Ahn

**Affiliations:** 1 Department of Aerospace and Software Engineering, Gyeongsang National University, Jinju 52828, Republic of Korea; 2024214517@gnu.ac.kr (J.D.); jaeho.kim@gnu.ac.kr (J.K.); 2 Department of AI Convergence Engineering, Gyeongsang National University, Jinju 52828, Republic of Korea; 2024214053@gnu.ac.kr

**Keywords:** multi-drone control, educational drones, centralized architectures, drone control station

## Abstract

**Highlights:**

**What are the main findings?**

- We present design processes, modeling artifacts, and a reference implementation for developing a drone control station to address the lack of team mission control among educational drones.
- The experimental result shows that the centralized architecture of the drone control station is feasible but limited when performing a team mission due to its hardware limitations. Still, the architecture is helpful for educational purposes.

**What are the implications of the main findings?**

- Drone software developers, educators, and students may adopt or reuse our presented design to control multiple educational drones.
- Low-cost educational drones can successfully execute team missions when paired with the appropriate communication strategy.

**Abstract:**

Educational drones have become increasingly important in education and research due to their affordability, user-friendly design and control, and potential use as tools in STEM (Science, Technology, Engineering, and Math) learning. For example, CoDrone EDUs are used to teach basic programming principles and drone control to high school or university students. As drones in real-world applications often collaborate to solve problems, controlling multiple educational drones in a team is crucial and beneficial for enhancing students’ problem-solving and design skills. However, these educational drones primarily rely on one-to-one control via a radio-frequency remote controller, and programming libraries for coordinating multi-drone missions are limited, posing challenges for students or developers in controlling them effectively. To address the lack of control in missions with multiple educational drones, we present a drone control station (DCS), featuring a centralized architecture that connects and controls various drones. We first develop scenarios and use cases that utilize multiple drones, specifying the system requirements. We then design conceptual models and architectures for the DCS. Next, we implement the DCS and evaluate whether it achieves the team missions. Experimental results show that the DCS with the centralized architecture is suitable for team missions with multiple educational drones. We expect the approach in our work to serve as a method for controlling multi-drone missions in an educational environment.

## 1. Introduction

The applications of Unmanned Aerial Vehicles (UAVs), or drones, equipped with cameras and sensors, have expanded across sectors such as education, military operations, facility management, and entertainment. In academic and research settings, educational drones such as the CoDrone EDU or Tello EDU are used due to their affordability, user-friendliness, and adaptability for experimental purposes. An educational drone is usually shipped with a single remote control. It typically relies on one-to-one control through a radio-frequency remote controller. This configuration provides a simple, cost-effective solution for indoor flight experiments that do not require high-end specifications or complex control systems [1]. For example, the CoDrone EDU provides support for individual operation, such as taking off, rolling (moving left or right), pitching (moving forward/backward), yawing (rotating left or right), throttling (moving up/down), or landing, making it ideal for basic programming education and simple flight tasks. Recently, interest in multi-drone missions, such as flying in team formation, has driven the need for scalable, synchronized control mechanisms [2,3], where reliable control and responsiveness among the drones are the goals [4].

Similar to educational drones, commercial or military drones are shipped with a remote control (hardware), containing ground control station (GCS) software. The GCS provides user interfaces and services for human operators to control a single enterprise or military drone but offers limited services for controlling multi-drone missions. In this regard, to control multiple educational, commercial, or enterprise drones, developers need to design and develop customized GCS software. However, the lack of supporting libraries and architectural designs for educational drones, such as CoDrone EDU, creates crucial limitations in controlling multi-drone missions. Educational drones also have low communication abilities, which makes it challenging to control multi-drone missions. This constraint underscores the need for an adaptable and scalable GCS that is not only efficient but also tailored to the capabilities of low-cost educational drones [5]. Without such a GCS, future educational and research applications may remain limited to single-drone use, restricting scalability, collaborative missions, and the advancement of multi-drone experimentation in academic and practical domains.

To address the lack of control in missions with multiple educational drones, we present a novel development process for a drone control station (DCS) system tailored to RF (radio-frequency) educational drones, from requirements elicitation and analysis to system design, implementation, and evaluation. We first develop scenarios and use cases using multiple drones while specifying system requirements. We then design conceptual models and an architecture based on the requirements. Next, we implement and evaluate the DCS centralized architecture for controlling multiple drones, with a focus on scalability. While developing the DCS, we adopt an object-oriented approach (OOA) to define and organize subsystems, components, and objects.

This work contributes key conceptual modeling artifacts, including scenarios, use cases, class diagrams, and architectural diagrams for controlling multiple educational drones. This work also contributes to a concrete layered architecture for classroom multi-drone control. This work can be adopted to develop a more advanced educational or commercial DCS. This paper highlights the trade-offs between simplicity and scalability, demonstrating that even low-cost educational drones can successfully execute coordinated missions.

This paper is structured as follows: Section 2 presents key concepts and background research, Section 3 describes the drone control station for multi-drone missions, and Section 4 introduces related work. Section 5 initiates the discussion, and Section 6 summarizes the key research work and its contributions.

## 2. Concepts and Background

We describe important concepts in the following section to provide an understanding of educational drones, multi-drone applications, and ground control stations.

### 2.1. Educational Drones

Currently, a wide range of educational drones are available on the market, each offering different capabilities depending on specific learning goals. A few of the educational drones available include the CoDrone EDU [6], Parrot Mambo Fly [7], DroneBlocks [8], the Crazyflie Drone [9], and DJI Tello EDU [10,11], all of which support programming in Python, Scratch, Blockly, and Swift. These drones are specifically designed for educational purposes [12], enabling students to program or experiment with autonomous flight control [11,12,13]. These programming features make drones valuable resources for classroom teaching and research applications related to swarm coordination, sensor integration, and basic autonomous behaviors [14,15,16]. Surveys indicate that drone-based learning enhances student engagement, interdisciplinary understanding, and computational thinking skills [17].

**Figure 1:**
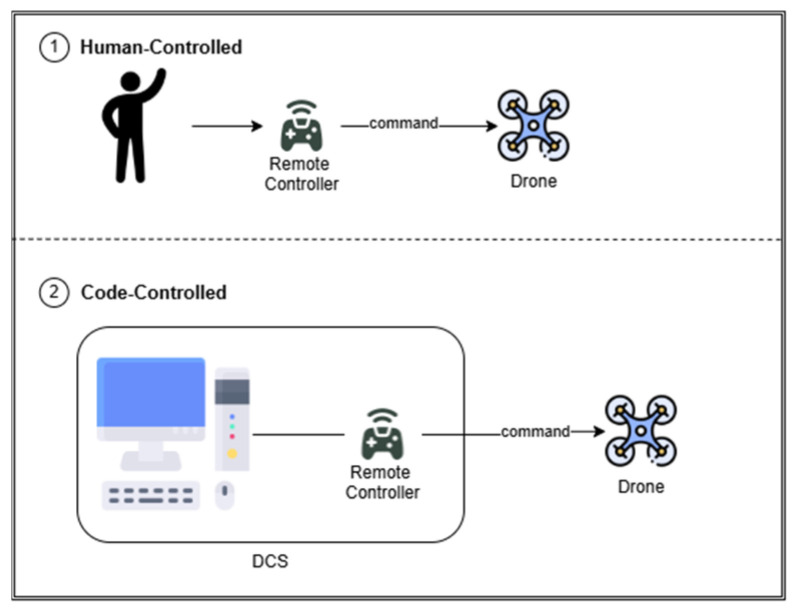
Drone control modes on CoDrone EDU.

CoDrone EDU, developed by Robolink [18], is an educational drone that supports programming languages such as Python via its dedicated CoDrone EDU library. Its affordability, simplicity, and easy learning tools make it ideal for educational purposes. The drone has a lightweight frame with four rotors; an integrated microcontroller; and sensors including a gyroscope, accelerometer, barometer, and infrared proximity detectors. It is also equipped with an RGB (Red, Green, and Blue) LED, a small speaker, and expansion module ports. Communication occurs over a 2.4 GHz RF link via a dedicated controller, meaning that each drone can connect to only one controller at a time, which poses challenges for multi-drone coordination. Figure 1 illustrates how the CoDrone EDU is controlled: an individual can operate it directly with a remote control (human-controlled) or through programming (code-controlled).

### 2.2. Applications Using Multiple Drones and Their Environment

Multi-drone missions have been adopted across various domains such as manufacturing and warehouse automation [19]. Fleets of drones are used for inventory monitoring [20] and for coordinating material-handling tasks. In the surveillance and security domain, multi-drone teams enable persistent monitoring, wide-area tracking, and rapid response in both indoor and outdoor environments [21,22]. Agriculture is another prominent domain in which coordinated drones perform crop monitoring [23], soil analysis, and synchronized pesticide spraying to reduce labor costs and improve precision [24,25].

Multi-drone deployment is also central in military applications, where coordinated UAV teams support reconnaissance, target tracking, and autonomous mission execution [26]. Entertainment domains, including drone light shows and interactive performances [27], demonstrate highly synchronized multi-drone control. These diverse applications underscore the growing importance of developing a reliable and accessible multi-drone control system, particularly in the education sector.

### 2.3. Ground Control Stations

A ground control station (GCS) serves as the primary command and control system for a UAV. The GCS serves as a link between the human operator and the drone, enabling control and mission management. GCSs may be stationary or mobile and typically include software that allows operators to set mission parameters, monitor real-time drone performance, review telemetry data, and, if necessary, control payload data [28]. In multi-drone settings, the GCS must manage multiple communication channels simultaneously, coordinate command distribution, and process telemetry from various sources. GCSs are essential for enhancing the reliability, efficiency, and safety of UAV missions, especially those involving multiple drones with limited onboard autonomy. Following the basic GCS concept, this paper refers to the drone control station (DCS) as a set of remote controls connected to an individual drone via radio-frequency and linked to a computer via USB.

## 3. Drone Control Station for Multi-Drone Educational Missions

We design a drone control station (DCS) with an object-oriented approach to address the lack of control in missions with multiple educational drones. The DCS consists of numerous software components, where the missions are defined, and the drone’s remote control establishes the connection with the drone. For drones with limited communication capabilities, the DCS needs to be designed and implemented with physical and software strategies to control multi-drone missions. The following sections present essential processes, concepts, and models for developing the DCS.

**Figure 2:**
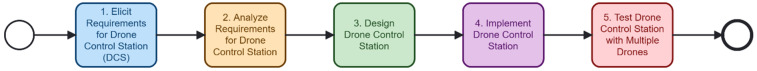
The overall process of developing the DCS.

To develop the DCS, we design a high-level development process, as shown in Figure 2. Developers may perform the process incrementally or iteratively, but we show the sequential process for simplicity. The process begins with the elicitation of system requirements, where functional and non-functional needs are gathered while considering stakeholders’ problems and goals [29]. These requirements are then analyzed to identify the initial system design. Based on this analysis, the DCS architecture and components are designed in the third step. The fourth step involves implementing the designed control station, which manages mission control and drone coordination. Finally, the workflow concludes with testing the DCS using multiple drones to validate scalability, acceptability of the system behavior, and suitability for multi-drone missions [30].

### 3.1. Step 1: Elicit Requirements for Drone Control Station

**Figure 3:**
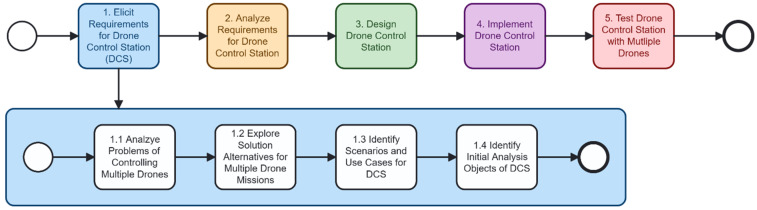
Requirements elicitation processes for the drone control station.

For the elicited requirements, we follow the steps in Figure 3. The activity is divided into four sub-activities. First, the problems associated with controlling multiple drones are analyzed to understand operational and communication limitations. Second, alternative solutions for coordinating multi-drone missions are explored. Third, representative scenarios and use cases for the DCS are identified to capture mission expectations and system interactions. Finally, the initial analysis objects of the DCS are defined, providing the essential concepts for subsequent modeling within the object-oriented design process.

#### 3.1.1. Analyzing the Problem of Controlling Multi-Drone Missions

The first step focuses on examining the inherent challenges associated with controlling missions with multiple low-resource educational drones. In Table 1, we list the limitations in communication protocols, issues relating to the lack of native multi-drone support, hardware constraints, and scalability issues that arise when coordinating multiple drones simultaneously.

#### 3.1.2. Exploring Solutions for Multi-Drone Missions

We explore alternative strategies and architectures for coordinating multi-drone missions to develop the DCS while accounting for the problems outlined in Table 1. Various architectural approaches are evaluated based on feasibility, complexity, and compatibility with educational drone platforms.

An analysis of several well-known frameworks was conducted to assess structures suitable for managing numerous educational drones, considering variables such as complexity, cost, and suitability for low-resource learning contexts (Table 2). The analysis also evaluated potential drone control strategies and justified the choice of one over another in multi-drone settings. These explored architectures included One-to-One RF Communication, a mesh network architecture [35], peer-to-peer (P2P) information exchange, cloud-based communication [36], a client–server architecture over TCP/IP, and a centralized architecture with a USB hub.

Due to its scalability and limitations in inter-drone communication, the One-to-One RF Communication approach, which is frequently used in educational drones, was not chosen. Similarly, mesh networks and P2P architectures were found to be inappropriate for educational contexts due to their high complexity in both hardware and software, which is not readily supported by simple drone platforms. Cloud-based communication [37] was excluded due to its reliance on internet connectivity and concerns about latency, which compromise real-time responsiveness. Although a client–server architecture is also a viable alternative, its implementation complexity makes it more suitable for future work. The centralized DCS with a USB hub configuration was chosen for its simplicity, cost-effectiveness, and classroom feasibility, despite limitations in dynamic task flexibility due to port constraints. We also performed a trade-off analysis for the design goals [38].

#### 3.1.3. Identifying Scenarios and Use Cases for DCS

In the third step, representative scenarios and use cases for controlling multiple drones are defined. The scenarios and use cases intend to capture how the DCS will interact with drones and human operators during multi-drone missions. Table 3 describes possible multi-drone mission scenarios that can be executed by drones with support from the DCS. It provides a brief description of each scenario, along with the key actions and interaction patterns that may occur within them.

**Figure 4:**
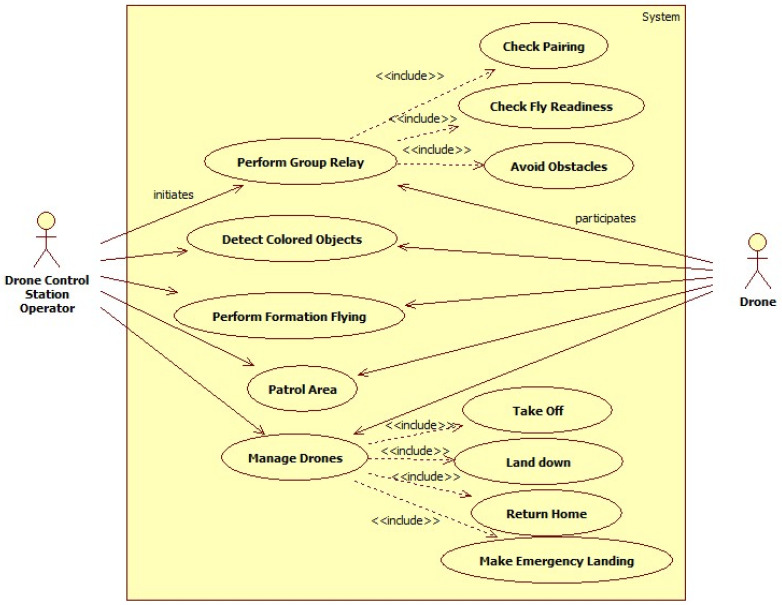
Use case diagram of the DCS system.

Building on the previously described scenarios, Figure 4 presents a use case diagram, which identifies the two primary actors interacting with the system: the operator and the drones. The diagram also includes additional use cases that support proper structured drone control within the DCS.

The operator initiates high-level drone missions. Each of these mission-level use cases involves multiple drones responding to commands issued by the DCS. Lower-level drone behaviors, such as *Take Off, Landing, Return Home, and Make Emergency Landing use cases* are modeled as included use cases, indicating their reuse across different mission workflows. Additional safety and readiness functions, *Check Pairing, Check Fly Readiness, and Avoid Obstacles use cases,* are included to ensure reliable operation before and during missions.

To better understand the use case diagram, we describe two use cases in the text, focusing on the flow of events and the interactions between the system and the main actors. In Table 4, the “perform team relay” use case is described, with the actor and system actions presented in different columns. The operator defines the relay parameters, and the DCS manages the takeoff order, flight to the turning point, and landing of each drone. Mission timing is recorded, and upon completion by the last drone, the DCS confirms successful execution while maintaining safety.

In Table 5, the “Detect colored objects” use case is described. It describes how a DCS operator initiates a multi-drone mission to detect a specific colored object. The operator configures the target color and drone team through the DCS, which then sends the detection command. Drones collaboratively scan the area, and once any drone detects the object, the DCS is notified, and the mission is halted for the remaining drones. A successful detection message is then displayed.

#### 3.1.4. Identifying Initial Analysis Objects of DCS

**Figure 5:**
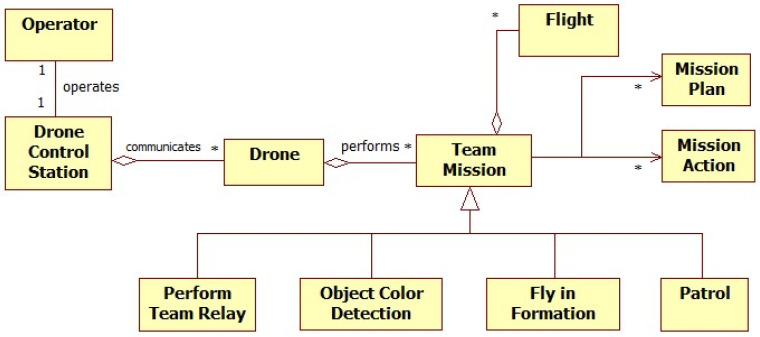
High-level class diagram of the DCS system.

In this step, we identify the initial analysis objects that serve as the conceptual basis for the DCS operations, with the identified problems, scenarios, and use cases. These analysis objects are the primary entities in the DCS application, including operators, drones, team missions, flights, mission plans, mission actions, and mission scenarios. We also identify the essential semantic relationship, such as ‘performs’, and express the association multiplicity as an asterisk (*) between the analysis objects. The high-level UML (Unified Modeling Language) class diagram in Figure 5 illustrates the system’s main components and their relationships. This object identification process bridges the gap between requirements and system design, laying the groundwork for object-oriented modeling.

### 3.2. Step 2: Analyze Requirements for Drone Control Station

**Figure 6:**
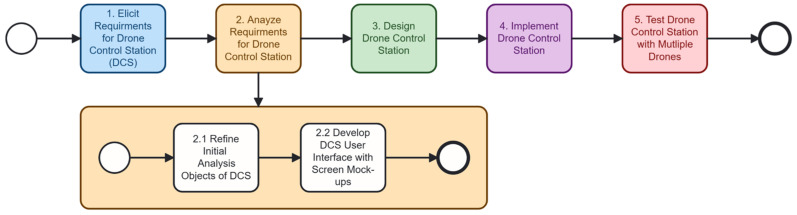
The requirements analysis process for the drone control station.

Figure 6 illustrates the second phase of the DCS development process, which focuses on refining and expanding the requirements analysis models. Step 2.1, Refine Initial Analysis Objects of DCS, revisits the preliminary objects identified in the previous phase and updates them based on an increased understanding of the system requirements, interactions, and constraints. Step 2.2, Develop DCS User Interface with Screen Mock-ups, establishes the preliminary user interface design through low-fidelity prototypes and operator support requirements.

#### 3.2.1. Refining Initial Analysis Objects of DCS

This step includes refining the initial analysis objects as we precisely define the information exchanged among the operators and DCS, which provides the foundation for the system design. By converting the original set of conceptual entities into specific analysis objects that accurately capture the elements required for a concurrent team mission, this technique goes over abstract requirements. For example, the concepts of “Team Mission, Flight, Mission Plan, Mission Action, and Scenarios” evolved into design-level components that support the operational behavior of the drone control station (DCS) and help refine and organize the system.

All mission execution-related components, such as the Team Mission object, are refined to encapsulate mission coordination and multi-drone synchronization logic. The Mission Plan and Mission Action objects are expanded to include route planning and execution, as well as message-handling responsibilities required for real-time operations. The Flight object is refined to represent the drone-specific state, telemetry integration, and command execution behavior. All low-level operational orders (including takeoff, motion, and landing) intended for specific educational drones are handled by the Drone Controller object. To enable prompt responses to events, the Scenarios object is extended to include operational rules, event conditions, and response handling for mission time situations. Thus, by modeling their sub-components and their clear linkages, the complete multi-drone mission control architecture is built to describe detailed system components, demonstrate the control hierarchy, and communicate the essential data during drone mission execution. Therefore, this refinement takes the abstract analysis objects and translates them into concrete objects, including boundary, control, and entity objects. These analysis objects enable mission execution, coordination, and system behavior within the DCS.

In addition to making the structural dependencies within the DCS clear, this thorough object identification and refinement process provides a precise blueprint for later dynamic and structural modeling using object-oriented techniques, successfully bridging the gap between high-level requirements and the final system architecture.

#### 3.2.2. Developing Initial DCS User Interface with Screen Mock-Ups

Analyzing and building an initial user interface (UI) for the DCS is a critical step in ensuring that operators can manage multiple educational drones safely, intuitively, and efficiently. At this initial stage of the requirements analysis, the focus shifts from high-level architectural decisions to the concrete representation of how users will interact with the system. Screen mock-ups serve as preliminary visual prototypes that illustrate functional elements of the DCS. These mock-ups enable early usability evaluation and help validate whether the interface aligns with the system’s operational requirements. By modeling the key interface components, such as mission configuration panels, drone status indicators, and command buttons, the design process ensures a cohesive user experience that supports the execution of multi-drone missions in a controlled and educational environment. In this step, we use generative AI, Stitch with Google [39], for prototyping the user interface of the DCS. We enter the use cases into Google Stitch and receive possible UI mock-ups. After the initial mock-up, we refine the generated mock-ups until they achieve a satisfactory interface.

Using generative AI for UI prototyping offers benefits, as it can make abstract ideas concrete, enabling conceptual system requirements and use cases to be translated into visual interface representations. These tools facilitate communication between designers and users, support early validation of functional assumptions, and accelerate the iterative design process. Additionally, the tool reduces the technical effort required to produce initial mock-ups, enabling researchers and students to focus on interaction logic rather than low-level design details.

**Figure 7:**
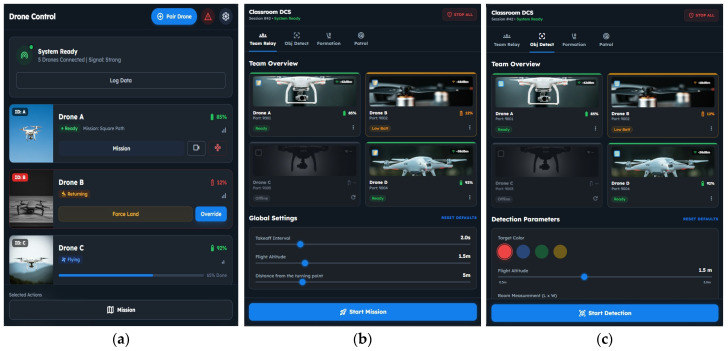
User interface. (**a**) Main window. (**b**) Team relay tab view. (**c**) Object color detection tab view.

Figure 7a presents the main window of the DCS. This interface provides an overall view of the system status and connected drones, including connectivity strength, battery levels, and current flight states. In this window, the operator can pair drones, monitor individual drone readiness, and issue basic commands such as mission assignment, forced landing, or override actions. The main window serves as the central entry point for managing the fleet of drones and initiating mission-level operations.

After clicking the “Mission” button in the main window, the operator is taken to the subsequent mission-specific windows. These interfaces are dedicated to configuring and executing coordinated multi-drone missions; for example, Figure 7b shows the team relay tab view. In this tab, the form for takeoff interval, flight altitude, and distance from the turning point can be customized, and the operator can select between the connected drones. Figure 7c shows the object color detection tab view, where the operator can choose parameters such as target color, flight altitude, and room measurements. It is also crucial that each mission-specific tab includes a “Stop all” button to ensure a safe flight. Together, these windows illustrate a structured workflow in which high-level control begins in the main DCS interface and transitions to specialized mission configuration screens.

### 3.3. Step 3: Design Drone Control Station

**Figure 8:**
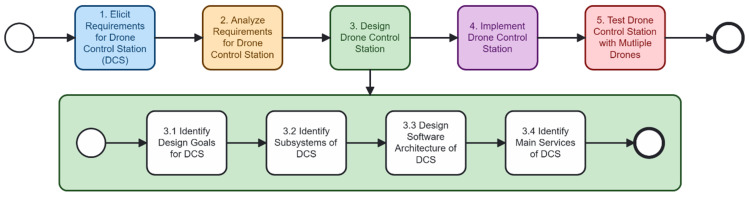
Design process of the drone control station (DCS).

Figure 8 presents the design activities involved in defining the architecture of the drone control station (DCS). The process begins with Step 3.1, Identify Design Goals for DCS, where the main objectives guiding the system’s development are identified. Next, Step 3.2, Identify Subsystems of DCS, decomposes the system into major functional components, allowing for more precise separation of responsibilities and more manageable design units. In Step 3.3, Design Software Architecture of DCS, these subsystems are structured into an architectural model that specifies component interactions and design constraints. Step 3.4, Identify Main Services of DCS, details the core functionalities that the system must provide to support multi-drone operation, including mission control, communication handling, and alert management.

#### 3.3.1. Identifying Design Goals for DCS

This first activity establishes the fundamental design objectives that the drone control station (DCS) must achieve. Based on earlier analysis, our design goals focus on enabling reliable coordination of multiple low-capability educational drones, supporting safe mission execution, and selecting communication mechanisms that operate effectively under constrained conditions. These goals of reliability, scalability, safety, and performance provide the guiding framework for the entire design process.

#### 3.3.2. Identifying Subsystems of DCS

**Figure 9:**
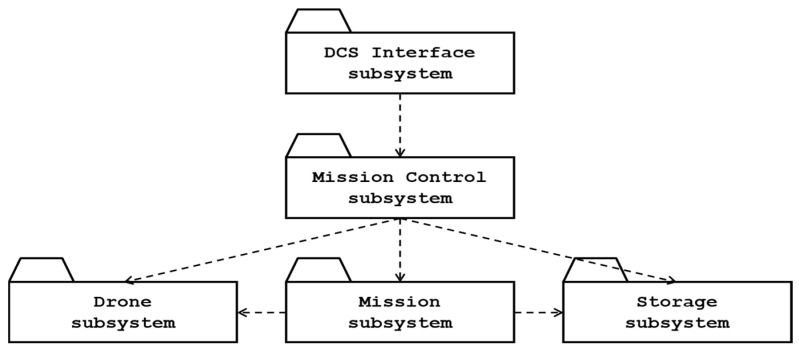
Subsystems of drone control station.

Identifying the subsystems of the DCS is a critical step. Based on the functional and non-functional requirements and design goals defined in earlier stages, the DCS system can be decomposed into DCS interface, mission control, mission, communication, and storage subsystems, as shown in Figure 9. Here, the dotted arrows denote a dependency. For example, the DCS Interface subsystem depends on the services of the Mission Control subsystem.

**The drone control station interface subsystem** provides user interfaces through which the operator interacts with the DCS system. It includes the main control station window and specialized interfaces for mission types such as team relay, object detection, formation flying, and patrol missions. This subsystem depends on the mission control subsystem and ensures intuitive access to mission configuration and control commands.

**The mission control subsystem** gets mission inputs or commands from the DCS interface subsystem and communicates with the mission, drone, and storage subsystems. This mission control subsystem handles initial mission setup, scheduling, drone pairing, and safety checks across multiple drones. This subsystem initiates a specific mission by sending a mission request to the mission subsystem. This subsystem also provides common drone functions, including obstacle avoidance, maintaining a safe distance, drone takeoff, and emergency landing. This subsystem directly interacts with various drone hardware components through communication protocols and stores critical mission data in the storage subsystem.

**The mission subsystem** encapsulates specific mission services. The mission subsystem provides services, including mission plans, flight information, mission tracking for the team relay, formation flying patterns, patrol strategies, and object detection. This subsystem also coordinates multiple drone flights, synchronizes them for the specific mission, and translates mission plans into direct drone commands.

**The drone subsystem** provides basic operations for managing specific educational drones with the native library. Educational drones enable basic flight operations, including takeoff, rolling (moving left or right), pitching (moving forward/backward), yawing (rotating left or right), throttling (moving up/down), and landing.

**The storage subsystem** manages persistent mission objects. This subsystem also manages the DCS’s system configurations. The storage subsystem may depend on either the native file or the database system in the execution environment.

#### 3.3.3. Designing the Software Architecture of DCS

**Figure 10:**
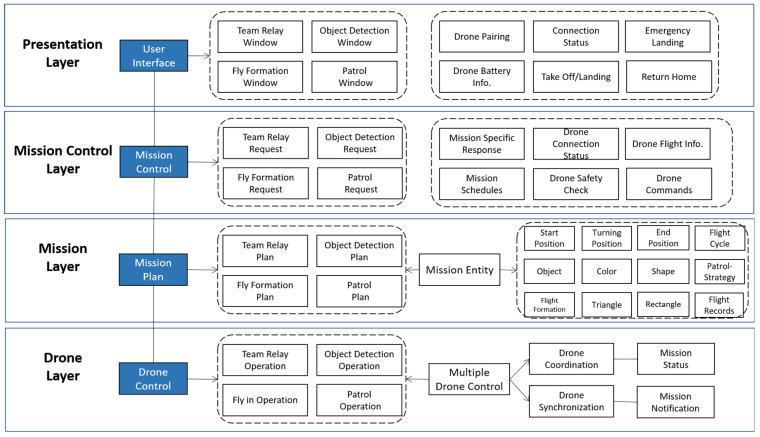
Multi-layered software architecture of DCS.

We design the DCS architecture based on the identified design goals and subsystems. We select a layered architecture to separate concerns across layers, ensuring modularity, scalability, safety, and maintainability for multi-drone mission operations. The layered architecture is illustrated in Figure 10, where the dash box represents logical components having similar functions, and the solid arrow represents an association.

**The presentation layer** contains boundary objects, including the DCS main window and mission-specific tab windows, including forms, buttons, and so on. The DCS operator can enter mission-specific information in this layer. For example, the operator may click the “Team relay” or “Object Detection” button. The operator can also manage DCS boundary conditions, including startup, shutdown, and exception handling.

**The mission control layer** manages the sequence of interaction with the DCS operator for multi-drone missions. This layer is responsible for building a mission request and then delegating it to the objects in the mission layer. The mission control object, also known as the mission controller, schedules multiple missions and monitors drone flight, as well as the mission’s outcome. This layer is also responsible for implementing safety checks, alerts, and notifications, as well as managing mission responses.

**The mission layer** plans a specific mission and executes it using multiple drones. This layer has mission-specific entity objects for multi-drone missions. The entity objects encapsulate fundamental data and methods that support mission planning, communication, and drone execution. Examples of entity objects include Times, Colors, Shapes, Formations, Patrols, and Emergency Landing Records.

**The drone layer** controls a specific drone through basic drone operations. This layer provides services for controlling drones, including drone connection, flight commands, flight sequences, setting or retrieving flight variables, and managing sensor data.

**The storage layer** handles the storage, retrieval, and query of persistent mission objects, such as the mission start and finish times. This layer also manages data for participating drone objects, the initial DCS settings, and multi-drone mission configurations.

**Figure 11:**
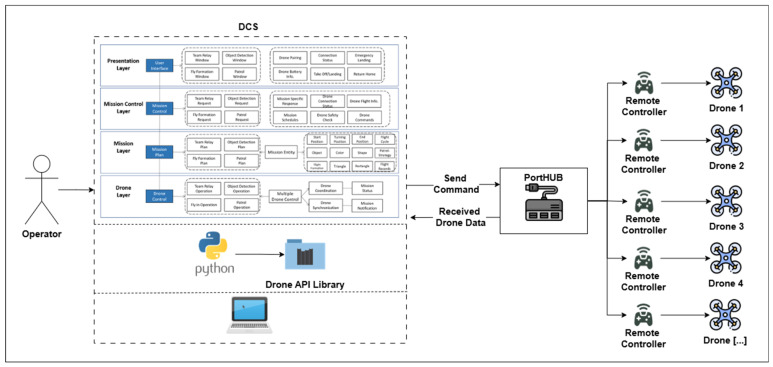
Centralized DCS controlling multiple drones.

As the DCS needs to control multiple drones to achieve specific objectives, we selected a layered, centralized architecture. The centralized architecture offers straightforward implementation and maintains full compatibility with the existing CoDrone EDU programming framework. The DCS executes a centralized Python script that uses the CoDrone EDU library on the drone layer to issue commands to each drone via the threading module. The program prepares each drone to receive the command and finish a coordinated operation. Drone identification and control are managed based on the specific USB port to which each remote controller is connected, facilitated using a USB hub, as shown in Figure 11.

Although this configuration allows for controlling several drones at once, it is inevitably limited by the physical constraints of available USB ports. The DCS transmits control commands to each drone via a USB port hub, enabling multiple drones to be connected.

#### 3.3.4. Identifying Main Services of DCS

This step identifies the essential services that the DCS subsystems need to provide to the upper layer. For example, the services in the presentation layer include drone registration and pairing, mission configuration, synchronized multi-drone control, telemetry visualization, emergency handling, and specialized mission capabilities such as formation flight or obstacle avoidance. Identifying these services clearly ensures that the subsystem’s functional scope is well defined and aligned with operational goals. We analyze the dependency between subsystems.

### 3.4. Step 4: Implement Drone Control Station

We implemented the drone control station using Python and the CoDrone Edu library. USB hubs, including the ipTIME UH308 of EFM Networks with eight ports (one power-only port + four USB 3.0 ports + three USB 2.0 ports), were used in series to connect to a single main PC for centralized control and configuration; an external power supply was not used. The hubs enabled multiple drones to be attached to a single computer simultaneously, and an individual CoDrone EDU unit was connected via a separate USB controller. With the help of the standard libraries, this setup enabled the execution of central commands, in which all drones received orders directly from the central computer.

**Figure 12:**
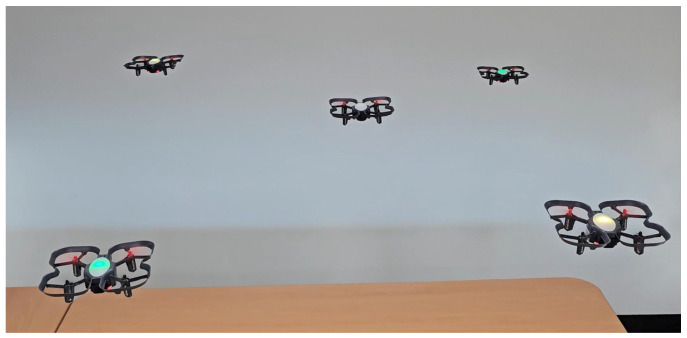
Drones performing the colored object detection mission.

For the centralized architecture, the Python script utilizes the “threading” package to ensure synchronized command execution across all connected drones. The program starts by identifying and listing all active serial ports for each CoDrone EDU device. Once all the drones are connected and registered, the user issues commands based on the intended mission. Multithreaded execution dispatches the command simultaneously, providing control with synchronized behavior. It ensures that all drones perform uniformly, regardless of whether they are connected to a single host computer. The source code used in the experiments is available on GitHub [40]. In Figure 12, five drones are connected and prepared to start the mission based on the colored object detection use cases.

### 3.5. Step 5: Test with Drone Control Station with Multiple Drones

#### 3.5.1. Testing the Scalability of DCS

To execute a multi-drone mission, the DCS must be connected to at least two drones; therefore, the command-and-control mechanism requires systematic testing and validation. Scalability tests were conducted to evaluate the system’s ability to maintain stable performance and reliable communication as the number of connected drones increased. These tests specifically examined how the proposed architecture handles increased command transmission and coordination demands under scaled conditions.

Initially, a single USB hub was used, with up to seven drones connected simultaneously. During this stage, the system remained stable, and all drones received and executed commands without issue. Communication between the DCS and each drone was consistent and reliable, indicating that the centralized setup is viable for a limited number of units.

When a second USB hub was connected in a daisy-chain (serial) configuration to the first hub to expand the number of ports, with 14 drones in total, intermittent connectivity issues began to occur. Drones frequently disconnected from and reconnected to the host computer, and command execution became less predictable. These issues worsened with the addition of a third hub, bringing the total to 21 drones, and the connection became increasingly unstable. Although some drones could still connect, the communication link was not reliable, and synchronized control was compromised. 

#### 3.5.2. Testing the Response Time

This experiment aims to evaluate the response time of the proposed DCS under increasing system load, specifically analyzing how communication is affected as the number of connected drones increases. Response time is the elapsed time between the operator issuing a command through the DCS interface and the moment the corresponding remote controller receives it and transmits it to the drone. This metric is critical for assessing the system’s responsiveness and practical usability in real-time multi-drone operations.

For this experiment, we connected multiple drone remote controllers in a serial (daisy-chain) configuration to a central host computer using USB hubs. The response time for basic flight commands was measured as the number of connected drones increased. To exemplify both use cases evaluated in this study, the experiment focused on the transmission of takeoff and landing commands, observing how the drones responded and behaved under different system loads. As shown in Table 6, the results indicate that the response time increased proportionally with the number of drones in the system. This behavior was attributed to the cumulative communication overhead introduced by the chained hub configuration.

**Figure 13:**
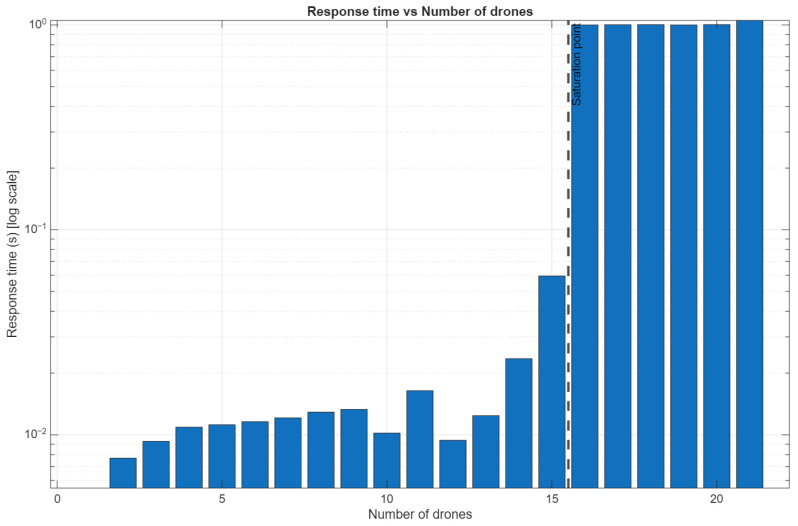
Response time as the number of drones is increased.

Figure 13 presents the scalability results from the response time experiment, illustrating how system performance evolves as the number of connected drones increases. The graph shows the relationship between the number of CoDrone EDU units and the measured command response time, capturing the delay between operator input at the DCS and each drone’s command reception. As observed, response time increases progressively with system scale, highlighting the communication overhead introduced by the centralized USB-based architecture. The figure also shows a decrease in connectivity stability when additional hubs are introduced, providing empirical evidence of the practical scalability limits of the proposed setup.

**Figure 14:**
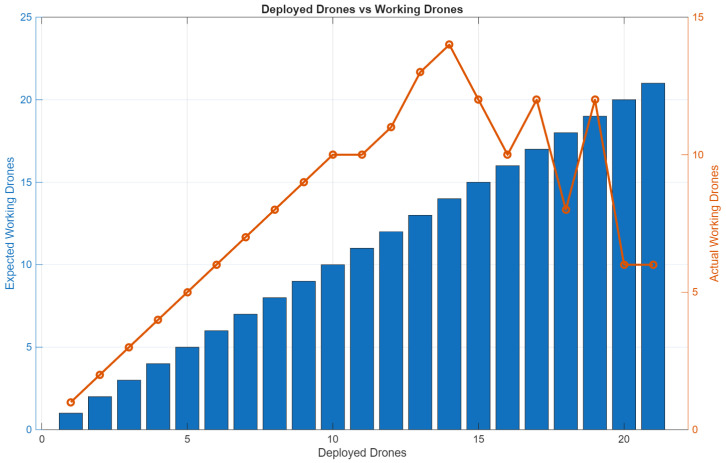
The number of actual working drones as the number of drones increases in the centralized architecture.

In addition, the experiment also evaluated connection stability. As the system scaled, connectivity issues emerged, including frequent disconnections and erratic behavior in the last controllers in the chain. These issues were caused by bandwidth limitations and power constraints of the USB hubs, which negatively impacted command delivery and synchronization. Furthermore, Figure 14 compares the expected number of operational drones with the actual number of drones that successfully maintained communication and executed commands. This expected-versus-actual analysis provided insight into how many drones were effectively able to “work” under each scaling condition. Therefore, alongside response time, the number of drones maintaining a stable connection versus the number of those experiencing failures was recorded, providing a complementary perspective on system robustness under scaling conditions.

Overall, the results demonstrate that, while the centralized architecture remains functional for a small number of drones, its performance degrades significantly as system load increases, highlighting the need for more scalable communication and control mechanisms for larger multi-drone deployments.

## 4. Related Work

In the drone control area, ground control stations (GCSs) are essential components in multi-UAV systems, providing mission planning, flight monitoring, and control functionalities. Prior research has examined multiple GCS architectures for coordinating UAV fleets for surveillance, exploration, mapping, and data collection [41]. Early studies introduced centralized GCS frameworks capable of issuing synchronized commands and aggregating telemetry in real time. At the same time, later work explored distributed and cloud-enabled models such as Cloud Station to enhance scalability and remote operation [42]. Although the hardware and educational applications of low-cost drones are well documented [43], less attention has been devoted to the control infrastructures or software platforms needed to manage them effectively in the classroom and collaborative environments [44]. Existing GCS platforms such as Mission Planner [45], QGroundControl [46], and UgCS are designed primarily for professional UAV operations and therefore lack interfaces and workflows suited for educational drones.

In the data communication domain, RF (radio-frequency) communication remains a reliable option for small educational multi-drone platforms despite its limited range and susceptibility to signal degradation [47], underscoring the importance of selecting communication methods based on mission requirements and system constraints. Other studies, such as those on network-aware GCS frameworks, propose latency reduction and reliability-enhancing strategies for ground–air communication [48]. Communication studies such as [49] highlight the trade-offs between mobile network-based (LTE/5G) and RF-based communication. While mobile networks offer greater coverage and higher throughput, they suffer from interference and handover instability. In contrast, traditional RF links, used predominantly in educational drones, enable low-latency, stable communication in short-range indoor settings. Multi-GCS collaboration models have been demonstrated in works such as [50], while broader surveys have identified persistent challenges related to operator workload, interface complexity, hardware integration, and system interoperability [51]. Although RF is widely used for communication between the DCS and educational drones via remote control, other advanced data communication protocols that use less computational resources or a modular firmware strategy may be selected for the DCS, depending on the system design goals, but work in this area is hard to find.

In the software development domain, the object-oriented approach (OOA), a de facto standard, enables drones to be conceptualized as autonomous components collaborating within a larger system framework, offering modularity and scalability through its principles of objects, classes, and structured interactions [52]. Use cases are used to elicit functional requirements by describing interactions between actors and the system, while class diagrams visually formalize the system’s structure by defining classes, attributes, methods, and relationships, forming a bridge from requirements to design. Systematic surveys of drone software engineering underscore the increasing importance of model-driven development, formal verification, and modular architecture for scalable UAV applications [53]. However, despite these advances, limited research has examined how the OOA can guide the structured design of DCS solutions tailored specifically for educational drones. Some DCS frameworks may adopt modular architectures, but they rarely demonstrate the use of requirements elicitation, use case modeling, or class diagram-driven design adapted to lightweight, low-resource platforms. Our work addresses this methodological gap in applying the OOA to the construction of scalable DCS architectures for drones.

## 5. Discussion

The study highlights that developing a drone control system (DCS) for educational drones requires a structured process encompassing requirements analysis, design, implementation, and experimentation. Creating a system that is both accessible and robust is challenging; additionally, existing ground control stations, such as QGroundControl and Mission Planner, are tools designed for UAVs with advanced onboard capabilities and standardized communication protocols, and their complexity, hardware requirements, and interface design make them difficult to use for developers and students without previous knowledge.

In contrast, the proposed DCS emphasizes collaborative missions and classroom applicability, though challenges remain such as ensuring stable communication among multiple drones, balancing simplicity and usability, and enabling students to understand and actively participate in the system’s design and operation. Furthermore, to support multi-drone coordination under constrained communication conditions (e.g., RF), the DCS is specifically designed and generally not suitable for existing professional platforms. The proposed DCS is more aligned with educational objectives, software engineering learning outcomes, and experimental multi-drone research using cost-effective platforms.

A centralized architecture, which is based on sending commands from a DCS over a USB port hub, is especially suitable for educational or classroom applications or controlled testing environments due to its simple implementation. There are hardware limitations, especially regarding the host computer’s maximum USB connection capacity and communication stability. When several hubs are linked together in series, the scalability is constrained. To some degree, the centralized architecture supports synchronized command execution and scalability; however, practical constraints imposed by network and hardware limitations remain.

Future work should explore other alternative architectures, AI-driven mission planning, enhanced simulation environments, and longitudinal studies to evaluate how students interact with the DCS over time, ultimately reinforcing its potential as a powerful tool for STEM education by fostering problem-solving, teamwork, and systems thinking. We plan to assign a DCS development project to undergraduate students in the upcoming semesters and evaluate whether the presented methods are helpful for students’ work.

The results encourage the use of inexpensive drone collections for classroom instruction, testing, and experimentation by providing researchers and educators with valuable insights. Furthermore, this study lays the foundation for future advancements in drone coordination systems, especially in educational settings with limited resources and accessibility, addressing the lack of multi-drone mission control in the educational drone domain.

### 5.1. Limitations

It is essential to consider the CoDrone EDU’s hardware constraints when performing the multi-drone scalability test. Additionally, it is cost-effective and suitable for educational settings; however, some limitations still exist with its use. The radio-frequency (RF) communication restricts the drone’s remote control range. The limited battery life supports only a limited flying duration, which can also cause inconsistent flight behavior, affecting stability and safety.

Similarly, the use of CoDrone EDU is limited to indoor environments, making it extremely sensitive to outdoor conditions and leading to unpredictable mission situations.

### 5.2. Threats to Validity

This study acknowledges several threats to validity that may affect the interpretation of the results.

- **Internal Validity:** The DCS concepts and architecture are based on the CoDrone Edu, as the authors have conducted projects and have development experience with this drone. If other educational drones are connected to this DCS, the DCS concepts and architecture may need to be updated to incorporate the functionality of these additional educational or small-to-medium-sized drones. Using a daisy-chain (serial) configuration to connect the hub results in an unstable connection between the drones.
- **External Validity:** Only CoDrone EDU units, educational drones with minimal sensing and connectivity capabilities, were used for DCS development and experimentation. The selected drones provide a controlled, consistent environment, making them suitable for verifying the proposed designs and team missions before scaling to more complex systems. The presented development process for DCS, however, needs to be adjusted for general reuse, as specific educational drones, e.g., CoDrone EDUs, are used. Some methods and features, such as GPS (Global Positioning System) tracking and drone display, are needed for a general drone control system.
- **Construct Validity:** The primary metrics used to evaluate mission success were the time required for execution, the mission’s scope, and the system’s behavior. These criteria were chosen because they accurately represent the effectiveness and dependability of multi-drone cooperation, although they may not capture all performance factors.

## 6. Conclusions

This study presents the object-oriented development process of a drone control station for educational multi-drone missions, beginning with the elicitation of requirements, followed by conceptual modeling, architectural design, implementation, and experimental evaluation; by systematically addressing communication limitations and scalability challenges through a centralized USB hub configuration, the proposed workflow not only demonstrates the feasibility of coordinating low-cost educational drones but also provides a replicable methodology that can guide future enhancements in multi-drone control systems for both academic and practical applications.

To overcome the drawbacks of their default one-to-one model, this study presents essential design concepts, including class diagrams and process models, for a drone control station. We also present a centralized architecture for the coordinated control of numerous CoDrone EDU units. Although it is easy to use and accessible for academic use, a centralized architecture is limited in scalability. A centralized architecture is feasible according to the tests, with hardware limitations being a significant drawback. A centralized architecture remains beneficial for simple applications and prototyping. It is better suited for academic and research use.

## Figures and Tables

**Table 1:** Identified problems for control of multiple educational drones.

Problem Category	Description of the Issue
One-to-One RF Communication [31]	Drones rely on a fixed 2.4 GHz RF link paired to a single controller, preventing simultaneous control of multiple drones from a single interface.
No Native Multi-Drone Support [32]	The ecosystem is designed for individual drone operation; the API and firmware lack built-in functions for team tasks, synchronized flight, or inter-drone communication.
Limited Communication Capabilities [33]	The drone cannot broadcast or receive data from other drones, restricting coordination strategies such as task sharing, distributed sensing, or collision avoidance.
Scalability Issues [34]	When connecting multiple remotes to a single station (e.g., via USB hubs), the connection becomes unstable, leading to delays, dropouts, or loss of control.

**Table 2:** Comparison of possible drone control approaches and selection rationale.

Architectures	Scalability	Simplicity	Limitations/Challenges	Selected/Not Selected and Why
One-to-One RF	--	+	No inter-drone communication; instructor workload	Not-Selected: Lacks coordination capabilities
Mesh Network	-	--	Hardware not supported in basic drone operations	Not-Selected: Incompatible with low-cost educational drones
Peer-to-Peer (P2P)	-	--	Poor control in the educational context	Not-Selected: Difficult to synchronize in real-time settings
Cloud-Based Communication	-	-	Requires internet access; latency issues	Not-Selected: Not suitable for real-time applications
Client–Server Architecture	++	-	Requires multiple PCs and a networking setup	Not-Selected: Enables synchronized multi-drone control.
Centralized with USB Hub	+	+	Limited by USB port count; less flexible for dynamic tasks	**Selected**: Cost-effective, simple to set up, and practical

Very Good ++, Good +, Bad -, Very Bad --.

**Table 3:** Possible multi-drone mission scenarios.

Scenario	Brief Description	Key Actions
Perform Team Relay	Students use three educational drones to fly from start -> turn -> finish using a custom DCS. Mission time determines ranking.	Initialize DCS, pair drones, set takeoff intervals and distances, start mission, coordinated takeoff and landing.
Detect Colored Objects	The team uses four drones to find a yellow card using onboard color sensors. Mission ends when one drone detects the card.	Set mission parameters, pair drones, start detection, coordinated flight, auto-halt, and return when color is detected.
Perform Formation Flying	Students fly drones in geometric formations (triangles, rectangles, pentagons). Updated DCS supports formation selection.	Define formation, update DCS UI, ensure flight safety, and execute stable formation flight.
Patrol Area	Three drones patrol while detecting obstacles using distance sensors. If a drone encounters an obstacle, notify the DCS and adjust the path. After a specific time, drones return to the start point.	Select avoidance mode, adjust parameters, detect obstacles, avoid, or return home.

**Table 4:** Perform team relay use case.

**Use case name**	Perform team relay
**Participating actors**	Initiated by the DCS (drone control station) operator Communicated with the drone team (with multiple drones)
**Flow of events**	1. The operator selects the “Team relay” tab on the DCS.
	2. DCS displays a “Team relay” form.
	3. The operator selects participating drones in the team relay and the distance to the turning point on the “Team relay” form, and sends the DCS a “Team relay” command.
	4. The DCS responds to the command, showing an “OK and starting the mission” message.
	5. The DCS sends the drone team the “Team relay” command, which includes the number of drones and the distance to the turning point. 6. The DCS records the start time of the “Team relay” and displays it in the DCS window.
	7. One drone in the team flies to the turning point, turns around the turning pole, and returns to the starting point. 8. The next drone flies and returns repeatedly, just like the first drone. 9. When the last drone completes and lands at the starting point, the DCS records the end time of the “Team relay” and displays it on the DCS window.
	10. The DCS displays a message indicating team relay completion in the DCS window.
	11. The operator confirms receipt of the team relay completion message.
**Entry condition**	● All the drones take off and hover, waiting for the mission command to be given.
**Exit condition**	● All the drones touch down at the mission location.
**Quality requirements**	● The drones take off and land safely. ● The command is sent from the DCS to the drones within 1 s.

**Table 5:** Detect colored object use case.

**Use case name**	Detect colored object
**Participating actors**	Initiated by the DCS (drone control station) operator Communicated with the drone team (with multiple drones)
**Flow of events**	1. The operator selects the “Detect colored object” tab on the DCS.
	2. The DCS displays a “Colored Object” form.
	3. The operator selects the color of the object for the drone team to detect in the “Colored Object” form and sends the DCS a “detect colored object” command.
	4. The DCS responds to the command, showing an “OK and starting the mission” message.
	5. The DCS sends the drone team the “detect colored object” command, including the object’s color.
	6. All the drones in the team fly over and perform the mission of detecting the colored object.
	7. When a drone in the team detects a colored object, it sends a detection message to the DCS and lands at its current location. 8. After receiving a message that the drone team has detected the colored object, the DCS sends a message to all other drones instructing them to land at their current locations. 9. The DCS displays “Colored object detected successfully” on the DCS window.
	10. The operator confirms the object detection message.
**Entry condition**	● All the drones take off and hover, waiting for the mission command to be given.
**Exit condition**	● All the drones touch down at the mission location.
**Quality requirements**	● The drones take off and land safely. ● The command is sent from the DCS to the drones within 1 s.

**Table 6:** Response time data.

Number of Drones	Response Time (Sec)	Working Drones	Additional Comments
1	0.0055	1	
2	0.0077	2	
3	0.0093	3	
4	0.0109	4	
5	0.0112	5	
6	0.0116	6	
7	0.0121	7	When connecting the second hub, the connection needs to be made again.
8	0.0129	8	
9	0.0133	9	
10	0.0102	10	
11	0.0164	10	The last connected drone is exhibiting erratic behavior.
12	0.0094	11	The last connected drone is exhibiting erratic behavior.
13	0.0124	13	When connecting the third hub, the connection needs to be made again.
14	0.0235	14	
15	0.0594	12	The drones connected to the third hub are disconnecting and are not receiving commands.
16	1	10	The drones connected to the third hub are disconnecting and are not receiving commands. Drones linked to port hub 2 show erratic behavior.
17	1.0023	12	The drones connected to the third hub are disconnecting and are not receiving commands.
18	1.0033	8	The drones connected to the third hub are disconnecting and are not receiving commands. Drones linked to port hub 2 are showing erratic behavior.
19	1.0003	12	The drones connected to the third hub are disconnecting and are not receiving commands.
20	2.0042	6	The drones connected to the third hub are disconnecting and are not receiving commands. Drones linked to port hub 2 show erratic behavior.
21	1.8524	6	The drones connected to the third hub are disconnecting and are not receiving commands. Drones linked to port hub 2 are showing erratic behavior.

## Data Availability

The original contributions presented in the study are included in the article; further inquiries can be directed to the corresponding author.
